# Evaluation of a 10% Urea Lotion for Xerosis: Clinical and Patient‐Reported Outcomes From a Prospective Study

**DOI:** 10.1111/jocd.70801

**Published:** 2026-03-10

**Authors:** Nemanja Maletin, Bojana Spasić, Zoran Golušin, Ivana Binić, Miloš Nišavić, Marija Ranđelović, Maša Golubović, Stefan Borocki, Lazar Marin, Iva Binić, Nikola Denda

**Affiliations:** ^1^ Faculty of Medicine University of Novi Sad Novi Sad Serbia; ^2^ Clinic of Internal Oncology Oncology Institute of Vojvodina Sremska Kamenica Serbia; ^3^ Clinic for Dermatovenerology University Clinical Center of Vojvodina Novi Sad Serbia; ^4^ Clinic for Dermatovenerology University Clinical Center Niš Niš Serbia; ^5^ Department of Dermatovenerology, Medical Faculty University of Niš Niš Serbia; ^6^ Department of Psychiatry, Medical Faculty University of Niš Niš Serbia; ^7^ Clinic for Eye Diseases University Clinical Center of Vojvodina Novi Sad Serbia

**Keywords:** dry skin, epidermal barrier, Prolom thermal water, skin hydration, urea, xerosis

## Abstract

**Introduction:**

Xerosis cutis is a common skin condition characterized by dryness, scaling, and pruritus due to impaired barrier function and reduced natural moisturizing factors. This study aimed to evaluate the clinical efficacy of a 10% urea lotion in adults with xerosis and assess participant satisfaction with the product.

**Methods:**

A prospective, single‐arm clinical study was conducted over 60 days in adults with confirmed xerosis. Participants applied a 10% urea lotion with Prolom thermal water twice daily. Skin hydration and elasticity were measured by corneometry and cutometry at baseline, day 14, day 30, and day 60. Subjective outcomes were assessed using a product satisfaction questionnaire and the Skindex‐16 scale. Data were analyzed using repeated measures ANOVA with *p* < 0.05 considered significant.

**Results:**

Significant improvements were observed in skin hydration (+55.3%) and elasticity (+55%), as well as reductions in redness, scaling, and itching (all *p* < 0.001). Skindex‐16 total scores improved by 61%, reflecting enhanced quality of life. The lotion was well tolerated, with no significant adverse effects, and participants reported high satisfaction and willingness to continue use.

**Conclusion:**

The 10% urea lotion with Prolom thermal water demonstrated significant clinical efficacy in improving hydration, elasticity, and overall skin condition in adults with xerosis over a 60‐day period. The treatment was well tolerated and associated with high patient satisfaction and a marked improvement in quality of life.

## 1 Introduction

Dry skin (xerosis cutis) is characterized by a deficiency of stratum corneum lipids and natural moisturizing factors (NMF) [1], leading to impaired barrier function. Clinically, xerosis is extremely common, affecting an estimated tens of millions (e.g., ~10 million in Germany) and roughly half of elderly individuals, and is often a primary dermatologic complaint [1, 2]. It causes tightness, scaling and pruritus, which can lead to excoriations and even infection, and significantly impact quality of life [3, 4].

At a pathophysiological level, xerosis reflects barrier dysfunction: normally the outermost SC prevents excessive transepidermal water loss (TEWL) [5]. When SC integrity is compromised, by reduced epidermal lipids or NMF (as in NMF‐deficient xerotic skin) [1], intrinsic aging changes in keratinization and lipid production [6], or environmental insults, TEWL increases and water‐holding capacity falls [5]. In older skin, altered keratinocyte maturation and reduced SC lipid content exacerbate these effects [6]. The result is the characteristic dry, rough, and often fissured skin of xerosis.

Urea, a small hygroscopic molecule, is a key component of NMF [7]. Topically applied, urea has multiple beneficial actions. It markedly increases stratum corneum hydration by attracting and retaining water, thereby reducing TEWL [7, 8]. At the 10% concentration, urea also exerts keratolytic effects by denaturing keratin hydrogen bonds to soften and shed scales [9]. Furthermore, urea promotes barrier repair by upregulating keratinocyte differentiation proteins (e.g., filaggrin, involucrin, transglutaminase‐1) and antimicrobial peptides [7], strengthening SC integrity. In sum, 10% urea lotion combines humectant, emollient and mild keratolytic actions that can rehydrate skin and restore barrier function.

In a comparative clinical study in elderly individuals, a moisturizer containing 10% urea demonstrated a significant improvement in skin hydration compared with a lanolin‐based petrolatum formulation, indicating superior humectant efficacy of urea‐containing preparations [10]. Furthermore, advanced instrumental imaging using near‐infrared confocal spectroscopic imaging (KOSIM IR) has shown that a 10% urea plus NMF formulation produces depth‐dependent increases in skin hydration and favorable volunteer perception when applied twice daily, confirming both objective and subjective benefits of urea‐based moisturizers [11]. A small open trial of ten older patients similarly reported marked improvement of senile xerosis after 10% urea cream application [12]. In diabetic foot xerosis, a 10% urea formulation produced faster clinical improvement than a glycerol‐based emollient [7]. In atopic dermatitis patients, both a 5% and a 10% urea moisturizer yielded similar significant reductions in SCORAD scores over 6 weeks [13], although subjects preferred the lower‐concentration formulation cosmetically. More recently, Lacarrubba et al. demonstrated that twice‐daily 10% urea cream significantly improved xerosis severity and itch within 7–14 days in elderly patients [14]. Anggraini et al. reported that a 10% urea cream increased skin capacitance (hydration) more than a 10% lanolin/petrolatum cream (increase 64.5% vs. 59.0%, *p* = 0.036) after 4 weeks in elderly xerotic skin [10]. Systematic reviews note that urea‐containing emollients (typically 5%–10%) consistently improve skin hydration and xerosis outcomes [7], though high‐quality head‐to‐head trials are few.

The Prolom 10% urea lotion was developed to combine urea's potent hydrating effects with the soothing properties of thermal spring water from Prolom Spa. The formulation is enriched with additional emollients and soothing ingredients to promote rapid rehydration and repair of the damaged epidermal barrier. This lotion is intended for use as an intensive moisturizing treatment for xerotic skin, leveraging both the humectant action of urea and the calming effects of the thermal water.

This study aimed to evaluate the clinical efficacy of a 10% urea lotion in adults with xerosis, as well as participant satisfaction. Outcomes included changes in hydration, elasticity, and self‐reported symptom improvement.

## 2 Methods

### 2.1 Study Design

This study was designed as a prospective, clinical, single‐arm, and longitudinal investigation aimed at evaluating the efficacy and tolerability of a 10% urea lotion in improving physiological and functional parameters of dry skin (xerosis).

The study was conducted over a five‐month period, from April 2025 to August 2025, including a 60‐day intervention phase. Measurements were performed at four time points:

- Baseline (T0, before treatment)
- Day 14 (T1)
- Day 30 (T2)
- Day 60 (T3, end of treatment)

At each visit, objective digital skin measurements and subjective questionnaires were obtained according to a uniform protocol. All assessments were performed by the same trained investigators to minimize inter‐observer variability.

### 2.2 Participants

A total of 47 adult participants with clinically confirmed xerosis of the lower legs were enrolled consecutively.

Inclusion criteria:

- Age 25–65 years.
- Visible clinical signs of dry, rough, or desquamating skin on the lower legs.
- Good general health without active dermatological disease.
- Willingness to comply with study procedures and scheduled visits.

Exclusion criteria:

- Known allergy or intolerance to any component of the tested product.
- Pregnancy or breastfeeding.
- Active inflammatory or infectious dermatoses.
- Use of systemic or topical corticosteroids, retinoids, or immunosuppressants within 30 days.

At baseline, demographic features, lifestyle factors (smoking, alcohol consumption, sun exposure), and Fitzpatrick phototype were recorded using a structured questionnaire.

All participants provided written informed consent prior to enrollment.

### 2.3 Tested Product

The tested product was a dermocosmetic body lotion containing 10% urea, designed for daily care of dry, rough, and desquamating skin. The formulation is based on natural Prolom water, an oligomineral thermal spring water originating from Prolom Spa in the Republic of Serbia, used as the primary aqueous phase of the product. The lotion combines urea as the principal active ingredient (10%), a well‐established humectant and keratoplastic agent, with a range of emollient, moisturizing, and skin‐protective components to support epidermal barrier function and improve cutaneous hydration.

The formulation includes a blend of occlusive and emollient lipids such as paraffinum liquidum and petrolatum, fatty alcohols (cetearyl alcohol, cetyl alcohol), and *Vitis vinifera* (grape) seed oil, which contribute to barrier restoration and reduction of transepidermal water loss. Additional functional ingredients include panthenol (pro‐vitamin B5) for epidermal regeneration, allantoin for soothing and epidermal repair, tocopheryl acetate (vitamin E) and ascorbyl palmitate (vitamin C ester) for antioxidant protection, alongside hydrogenated farnesene (hemisqualane) for improved sensorial properties and skin compatibility. The product is preserved using a combination of phenoxyethanol and ethylhexylglycerine, supported by citric acid for pH stabilization.

Collectively, these components are formulated to act synergistically to enhance skin hydration, softness, smoothness, and barrier function, while reducing subjective symptoms of dryness, roughness, and discomfort. The product is dermatologically tested and classified as a leave‐on formulation, intended for adult use.

Product safety, stability, and quality have been confirmed through an official cosmetic product safety assessment conducted in accordance with Regulation (EC) No. 1223/2009, applicable Serbian national regulations, and complementary toxico‐dermatological evaluations.

Products were provided by the sponsor in standardized clinical packaging.

### 2.4 Intervention and Application Protocol

Participants applied the 10% urea lotion to the anterior and lateral surfaces of both lower legs for 60 consecutive days.

Application protocol:

- twice‐daily (morning and evening),
- on clean and dry skin,
- applied in a thin layer and massaged until absorbed.

Participants were instructed not to use any additional topical products on the test area during the study period and not to apply any product on the day of scheduled assessments.

Compliance was evaluated verbally at each visit and by monitoring product usage.

### 2.5 Standardization Procedures

To ensure accurate and reproducible instrumental measurements, all assessments were conducted under controlled environmental conditions.

Pre‐measurement standardization:

Before measurements, participants:

- avoided topical products for at least 12 h,
- cleaned the skin with lukewarm water only,
- gently dried the skin without rubbing.

A 15‐min acclimatization period was observed in a controlled environment:

- Temperature: 22°C ± 2°C
- Relative humidity: 45%–55%

This procedure minimized variability caused by environmental factors, perspiration, or transient hydration.

### 2.6 Measurement Sites and Assessment Protocol

Objective measurements were obtained at the central region of the lateral lower leg, bilaterally.

For each time point:

- three consecutive measurements were taken per site,
- mean value was used for analysis,
- bilateral values were averaged to obtain global scores.

All measurements were performed by the same examiner using the same device.

### 2.7 Objective Skin Assessment

Objective physiological and biomechanical parameters were evaluated using the Multi Skin Test Center MC 1000 (Courage + Khazaka Electronic GmbH, Germany), equipped with the following probes:

- Corneometer‐hydration (corneometric units).
- Cutometer‐elasticity (arbitrary units).
- Sebumeter‐surface lipids (μg/cm^2^).
- Mexameter‐erythema and pigmentation (index values).
- Visioscope PC 35‐high‐resolution digital imaging of surface morphology.

The MC 1000 software automatically recorded and stored measurements in an electronic database using anonymized participant codes.

### 2.8 Subjective Evaluation Instruments

#### 2.8.1 Urea Lotion Efficacy Questionnaire

All questionnaires were administered from day 14 (T1) onward. Participants evaluated:

- skin hydration
- elasticity
- roughness
- scaling
- itching
- overall satisfaction
- willingness to continue use
- willingness to recommend

Scores were recorded on a five‐point Likert scale, with higher values indicating greater improvement.

#### 2.8.2 Skindex‐16 Quality‐of‐Life Questionnaire

Quality of life was assessed using Skindex‐16, including three domains:

- Symptoms
- Emotions
- Functioning

Domain scores were calculated as arithmetic means; an overall score was computed as the average of all items.

### 2.9 Ethical Considerations

The study was conducted in accordance with the ethical standards of the institutional research committees and with the 1964 Helsinki Declaration and its later amendments. Ethical approval was obtained from the Ethics Committee of the University Clinical Centre of Vojvodina, Clinic of Dermatovenereology (Approval No. 00‐156/2025; date: 21 March 2025) and the Ethics Committee of the Faculty of Medicine, University of Niš (Approval No. 12‐3102‐1/2‐6; date: 3 April 2025). All participants provided written informed consent prior to enrolment.

### 2.10 Statistical Analysis

All numerical data were entered into an electronic database and analyzed using the Jamovi statistical software (version 2.5.0). Descriptive statistics included means, standard deviations, and ranges. To analyze changes over time, Repeated Measures Analysis of Variance (RM‐ANOVA) was employed. Data normality was tested using the Kolmogorov–Smirnov test. When assumptions of normal distribution were not met, the Friedman test was used as a non‐parametric alternative. All statistical tests were two‐tailed, and significance was defined as *p* < 0.05.

## 3 Results

Table 1 presents the demographic and lifestyle characteristics of the study participants. A total of 47 individuals were included, predominantly female (80.9%), with a mean age of 42.4 ± 10.3 years. The most represented age group was 25–35 years (37.8%). According to the Fitzpatrick classification, phototypes II (55.3%) and III (21.3%) were most common. Most participants were non‐smokers (59.6%) and reported occasional alcohol consumption (57.4%). Sun exposure was predominantly moderate (83.0%), while tanning bed use was uncommon (10.6%).

**TABLE 1:** Demographic and lifestyle characteristics of the study participants.

Characteristic	Category	*n* (%)
Sex	Male	9 (19.1%)
	Female	38 (80.9%)
Age (years)	Mean ± SD (min–max)	42.4 ± 10.3 (26–64)
Age group	25–35 years	17 (37.8%)
	36–45 years	11 (24.4%)
	46–55 years	11 (24.4%)
	56–60 years	6 (13.3%)
Fitzpatrick skin phototypes	I	9 (19.1%)
	II	26 (55.3%)
	III	10 (21.3%)
	IV	2 (4.3%)
Smoking status	No	28 (59.6%)
	Occasionally	7 (14.9%)
	Yes	12 (25.5%)
Alcohol consumption	No	20 (42.6%)
	Occasionally	27 (57.4%)
Sun exposure	Rarely	7 (14.9%)
	Moderately	39 (83.0%)
	Frequently	1 (2.1%)
Tanning bed use	Yes	5 (10.6%)
	No	42 (89.4%)
Occupational sun exposure	Yes	1 (2.1%)
	No	46 (97.9%)

Significant improvements were observed across all evaluated subjective skin parameters over the 60‐day application as period shown in Table 2. Dryness improved from 2.18 ± 0.75 at T1 to 2.87 ± 1.00 at T3 (*p* < 0.001), while hydration demonstrated a marked increase from 3.31 ± 0.79 to 3.84 ± 0.79 (*p* < 0.001). Skin elasticity also showed a steady upward trend (3.10 ± 0.55 to 3.56 ± 0.83, *p* < 0.001). Symptom burden associated with xerosis decreased over time, with improvements in overall symptoms (3.18 ± 0.75 to 3.44 ± 0.80, *p* < 0.001), scaling (2.92 ± 0.76 to 3.09 ± 1.01, *p* < 0.001), and itching (2.49 ± 0.90 to 3.04 ± 1.13, *p* < 0.001). Repeated‐measures ANOVA confirmed statistically significant time effects for all parameters (all *p* < 0.001), indicating consistent and clinically meaningful benefits of continuous 10% urea lotion application.

**TABLE 2:** Changes in subjective skin parameters during Prolom 10% urea lotion application over 60 days.

Parameter	T1 (Mean ± SD)	T2 (Mean ± SD)	T3 (Mean ± SD)	*p* ^a^
Dryness	2.18 ± 0.75	2.67 ± 1.01	2.87 ± 1.00	< 0.001
Hydration	3.31 ± 0.79	3.56 ± 0.76	3.84 ± 0.79	< 0.001
Elasticity	3.10 ± 0.55	3.37 ± 0.86	3.56 ± 0.83	< 0.001
Symptoms	3.18 ± 0.75	3.40 ± 0.78	3.44 ± 0.80	< 0.001
Scaling	2.92 ± 0.76	3.05 ± 1.01	3.09 ± 1.01	< 0.001
Itching	2.49 ± 0.90	3.02 ± 1.15	3.04 ± 1.13	< 0.001

*Note:* Values are presented as mean ± standard deviation (SD). T1 = day 14; T2 = day 30; T3 = day 60. All parameters were analyzed using repeated‐measures ANOVA, with statistically significant improvements observed across all timepoints (*p* < 0.001 for all comparisons).

Subjective evaluation in Table 3 demonstrated consistently high satisfaction with the 10% urea lotion throughout the study, with mean scores remaining favorable from T1 to T3 (3.82 ± 0.51 to 3.47 ± 0.47; *p* < 0.05). Participants also showed a strong intention to continue using the product and to recommend it to others, with already low baseline scores decreasing further over time, although these changes were not statistically significant due to ceiling effects. Safety assessments indicated excellent tolerability, with adverse‐effect scores remaining stable and minimal across all visits. Overall, the lotion was well accepted, safe, and positively rated by participants during continuous use.

**TABLE 3:** Subjective assessment of satisfaction, intention to continue use, recommendation, and safety of the Prolom 10% urea lotion.

Parameter	T1 (Mean ± SD)	T2 (Mean ± SD)	T3 (Mean ± SD)	*p* (RM‐ANOVA)
Satisfaction with effects	3.82 ± 0.51	3.40 ± 0.49	3.47 ± 0.47	< 0.05
Intention to continue use^a^	1.74 ± 0.36	1.49 ± 0.34	1.47 ± 0.33	n.s. (ceiling effect)
Recommendation to others^a^	1.56 ± 0.28	1.47 ± 0.27	1.44 ± 0.26	n.s. (ceiling effect)
Adverse effects	1.13 ± 0.08	1.05 ± 0.06	1.04 ± 0.04	n.s.

*Note:* Values are presented as mean ± standard deviation (SD). Lower scores on the marked items indicate more favorable responses (1 = “Yes, definitely”). T1 = day 14; T2 = day 30; T3 = day 60.

Abbreviation: n.s., non‐significant.

As shown in Table 4, Skindex‐16 scores improved significantly across all domains over the 60‐day follow‐up period (all *p* < 0.001). Symptom scores decreased by 61.5%, emotional impact by 59.3%, and functional impairment by 63.2% from baseline to T3. The total Skindex‐16 score also declined markedly (−61.3%), indicating a substantial improvement in overall skin‐related quality of life with continuous use of the 10% urea lotion.

**TABLE 4:** Changes in Skindex‐16 scores over the follow‐up period.

Domain	T0 (Mean ± SD)	T1 (Mean ± SD)	T2 (Mean ± SD)	T3 (Mean ± SD)	*p* (RM‐ANOVA)
Symptoms (items 1–5)	2.83 ± 1.02	2.18 ± 0.87	1.64 ± 0.74	1.09 ± 0.61	< 0.001
Emotions (items 6–12)	3.05 ± 1.11	2.31 ± 0.93	1.77 ± 0.72	1.24 ± 0.57	< 0.001
Functioning (items 13–16)	2.64 ± 0.94	2.01 ± 0.78	1.52 ± 0.63	0.97 ± 0.46	< 0.001
Total Skindex‐16 (0–6)	2.84 ± 0.98	2.17 ± 0.84	1.64 ± 0.69	1.10 ± 0.55	< 0.001

*Note:* Values are presented as mean ± standard deviation (SD). Lower scores indicate fewer symptoms and better quality of life. T0 = baseline; T1 = day 14; T2 = day 30; T3 = day 60. All domains showed significant improvement over time (*p* < 0.001).

Objective instrumental measurements demonstrated significant improvements from baseline across all skin parameters except sebum (Table 5). Redness and pigmentation decreased progressively, with reductions of 30%–35% by T3 (*p* < 0.001). Hydration and elasticity showed marked enhancement over time (*η* ^2^ = 0.467 and 0.524, respectively), reflecting substantial restoration of skin barrier function. Surface irregularities, including spot intensity and spot structures, also decreased significantly (*p* < 0.001), indicating notable textural refinement. Sebum levels fluctuated without meaningful change (*p* = 0.266). Overall, repeated‐measures ANOVA confirmed strong treatment effects across most physiological and morphological parameters.

**TABLE 5:** Objective instrumental skin measurements and longitudinal changes across the 60‐day follow‐up.

Parameter	T0 (Mean ± SD; min–max)	T1 (Mean ± SD; min–max)	T2 (Mean ± SD; min–max)	T3 (Mean ± SD; min–max)	*F*(df)	*p*	*η* ^2^
Sebum	0.72 ± 2.00 (0–10)	1.13 ± 2.09 (0–8)	0.68 ± 1.64 (0–8)	0.51 ± 1.41 (0–7)	1.33 (3138)	0.266	0.016
Redness	42.3 ± 11.8 (17–66)	37.3 ± 10.6 (15–60)	33.4 ± 9.64 (14–53)	29.6 ± 9.82 (9–50)	170.0 (3138)	< 0.001	0.172
Pigmentation	23.1 ± 7.91 (10–46)	19.3 ± 6.02 (6–33)	16.8 ± 5.32 (7–32)	15.2 ± 5.11 (4–30)	95.10 (3138)	< 0.001	0.191
Hydration	38.7 ± 8.06 (16–51)	46.4 ± 8.54 (28–72)	52.7 ± 8.51 (36–77)	60.1 ± 8.94 (45–80)	174.0 (3138)	< 0.001	0.467
Elasticity	48.3 ± 8.81 (25–74)	57.6 ± 8.42 (30–78)	67.4 ± 9.64 (35–86)	74.9 ± 11.4 (41–99)	191.0 (3138)	< 0.001	0.524
Spot	4.37 ± 1.53 (1.1–8.4)	3.40 ± 1.19 (0.9–6.3)	2.66 ± 1.10 (0.8–5.6)	1.80 ± 0.96 (0–5.0)	126.0 (3132)	< 0.001	0.376
Spot structures	41.4 ± 3.61 (35–55)	38.2 ± 3.41 (31–44)	35.3 ± 3.63 (29–44)	31.8 ± 3.95 (22–42)	174.0 (3138)	< 0.001	0.487

*Note:* Values are reported as mean ± standard deviation (SD) with minimum–maximum range. T0 = baseline; T1 = day 14; T2 = day 30; T3 = day 60. Effect size is expressed as eta‐squared (*η* ^2^). Lower redness, pigmentation, spot, and structural scores indicate improvement; higher hydration and elasticity indicate improvement. Sebum showed no significant change over time.

## 4 Discussion

Our study demonstrated a consistent and statistically significant improvement in skin barrier function parameters following twice‐daily application of a 10% urea lotion enriched with Prolom thermal water over a 60‐day period. The most pronounced effects were observed in skin hydration and elasticity. Objective measurements showed a 55.3% increase in corneometric hydration values, indicating marked rehydration of the stratum corneum. This finding aligns with the known humectant properties of urea, which enhance water retention in the epidermis and reduce transepidermal water loss. In parallel, cutometric analysis revealed a 55% improvement in skin elasticity, reflecting restoration of biomechanical properties likely mediated by improved hydration, barrier repair, and enhanced lipid content. These changes occurred progressively, with early improvements evident by day 14 and further gains through day 60, suggesting both rapid onset and sustained benefit. Together, these results confirm the strong moisturizing and biomechanical efficacy of the tested 10% urea formulation in adults with xerosis.

In addition to the marked improvements in hydration and elasticity, our study also showed consistent subjective improvements in skin roughness and dryness, confirming the visible and perceptible benefits of the treatment. These findings are in line with those reported by Schölermann et al., who demonstrated that a 10% urea lotion significantly increased hydration and reduced clinical signs of xerosis in elderly patients when compared to placebo‐treated skin [15]. Similarly, Gallinger et al. used advanced methods such as corneometry and near‐infrared confocal spectroscopy to confirm the superior moisturizing performance of a 10% urea cream enriched with natural moisturizing factors over its vehicle base [11]. While most existing studies did not assess elasticity directly, it is well established that improved hydration leads to better biomechanical skin properties, including increased suppleness and resilience. In our trial, the observed improvements in skin texture likely contributed to the measured enhancement in elasticity. Conversely, pigmentation levels remained relatively stable, as expected, since urea does not directly influence melanocyte activity or melanin synthesis. However, we did note an overall improvement in skin appearance, which may reflect a reduction in secondary inflammatory changes associated with chronic dryness. This observation aligns with the findings of Seifert et al., who documented a visible decline in erythema and lichenification after 4 weeks of urea treatment, suggesting decreased irritation and more even skin tone [16]. This is further supported by in vitro studies demonstrating that urea, even under reduced humidity conditions, helps maintain the structural stability of the stratum corneum and preserves skin permeability by interacting with both lipid and protein components of the barrier [17].

Subjective outcomes also improved significantly. Participants reported relief from uncomfortable xerosis symptoms, including itching, scaling, and the sensation of tight or dry skin. The Skindex‐16 score, a validated and widely used quality‐of‐life instrument in dermatology, showed over 60% improvement across all three domains (symptoms, emotions, and functioning), indicating substantial gains in daily functioning and psychological well‐being. This finding aligns with the methodological robustness of Skindex‐16, as demonstrated in the original validation study by Chren et al., which confirmed high internal consistency (*α* = 0.86–0.93) and strong responsiveness to changes in skin condition [18]. Our findings are consistent with those of Lacarrubba et al., who reported significant itch reduction (via VAS scale) and complete resolution of flaking on dermoscopic examination after 2 weeks of using a 10% urea cream [14]. No adverse events were noted in that study, and all participants rated the product positively, with approximately 70% giving it the highest rating (“excellent”) [14]. Similarly, Anggraini et al. found that twice‐daily application of 10% urea cream in elderly individuals resulted in complete resolution of itching after 4 weeks, along with a significantly greater increase in skin hydration compared to a lanolin‐based cream (*p* = 0.036) [10]. Notably, although both formulations alleviated xerosis symptoms in that randomized study, urea had a superior physiological effect on skin barrier function [10, 14]. Our study confirms that 10% urea not only effectively reduces symptoms like itching and dryness but also contributes to barrier restoration, which may be essential for long‐term symptom control. These findings support the broader use of 10% urea products as a therapeutic option that combines clinical efficacy with excellent tolerability and patient satisfaction.

The formulation of a moisturizing product has a significant impact on user experience and overall treatment success. Urea‐based products are generally considered safe and well tolerated in xerosis treatment; however, higher concentrations of urea may occasionally cause transient stinging, particularly on damaged or inflamed skin [7, 8, 9, 19, 20]. Bissonnette et al. compared a 10% urea lotion with a newly developed 5% urea formulation in patients with atopic dry skin and found that both products resulted in similar clinical improvement (approximately 19% SCORAD index reduction over 6 weeks). Nevertheless, participants tended to prefer the 5% formulation due to better subjective tolerability [13]. This suggests that factors such as texture, absorption rate, scent, and mild stinging may influence patient preference, even when therapeutic efficacy is equivalent.

In our study, the 10% urea lotion was very well tolerated; most participants were satisfied with the feel on the skin and reported no adverse reactions. These findings are supported by Anggraini et al., who showed that a 10% urea cream was not associated with increased side effects compared to traditional ointments (lanolin in petrolatum), and even caused less perceived stickiness, though the difference was not statistically significant [10]. These observations are consistent with findings from a randomized double‐blind study by Lodén et al., which compared the effects of a 4% urea cream and a 20% glycerin cream in patients with atopic dermatitis. While both formulations produced similar improvements in skin dryness, the urea cream was associated with a significantly higher incidence of smarting sensations, reported by 24% of users compared to only 10% in the glycerin group (*p* < 0.0006), particularly when applied to excoriated or sensitive skin [21]. These results highlight that tolerability can differ between formulations, even when their efficacy is comparable. In other words, a well‐formulated urea cream can provide a high level of comfort during use, sometimes even superior to classic emollients, while delivering clear clinical benefits that users notice directly. Overall patient satisfaction in both our study and comparable trials was high [10], as reflected in the strong willingness to continue product use, indicating that the benefits (such as rapid relief from dry and itchy skin) outweigh minor drawbacks (such as scent or mild stinging) from the user's perspective.

Additionally, the specificity of the tested formulation is supported by the inclusion of Prolom thermal water as the formulation vehicle. Prolom water is an oligomineral, sodium‐bicarbonate thermal water with high balneological value, traditionally used in dermatologic and rehabilitative settings [22, 23]. Mineral and thermal waters have been shown to support skin hydration, barrier recovery, and symptom relief in various inflammatory and xerotic dermatoses, primarily through their physicochemical properties and mineral composition rather than direct pharmacologic activity [24]. Prolom thermal water contains metasilicic acid, a bioavailable form of silicon, which has been associated with skin conditioning and hydration support in balneological contexts [22, 23, 25]. While direct effects on dermal collagen synthesis were not evaluated in the present study, the mineral composition of the water may indirectly contribute to improved skin surface characteristics by supporting epidermal hydration and barrier integrity. In addition, the high alkalinity of Prolom water (pH approximately 8.8–9.2) and its ionic profile may influence water‐binding capacity and stratum corneum hydration, thereby providing a favorable environment for barrier repair [22]. Importantly, within the context of the present study, Prolom thermal water should be regarded as a supportive vehicle rather than an independent active agent. The observed improvements in skin surface parameters are most plausibly attributable to the established pharmacodynamic effects of urea on corneocyte cohesion, natural moisturizing factor replenishment, and stratum corneum hydration, with the mineral water base potentially enhancing tolerability and sustained hydration performance of the formulation [22, 23, 24, 25].

Our findings are consistent with the broader dermatological understanding of urea's role in skin care. In a recent observational study involving 59 elderly individuals with senile xerosis of the lower legs, treatment with a 10% urea lotion over 4 weeks led to significant improvement in all clinical signs of dry skin, including erythema, lichenification, and scaling, along with enhanced barrier function and reduced transepidermal water loss [16]. In parallel, patients reported noticeable symptom relief as early as day 7, and by the end of the study, most indicated clear improvements in their quality of life as a result of reduced discomfort [16]. Furthermore, a literature review conducted by Parker and colleagues highlighted that urea‐based formulations (typically 10%–25%) yield impressive results in the treatment of pronounced xerosis, outperforming standard emollients lacking active ingredients [26]. Even in more severe cases, such as extremely dry and thickened skin on the feet, 10% urea emulsions showed better outcomes than petrolatum alone, and increasing the concentration of urea (up to 20%–40%) can further accelerate the softening of hyperkeratotic areas [26]. Taken together with the results of our study, these findings confirm that 10% urea is an effective and evidence‐based option for managing dry skin and treating xerosis of various origins. Beyond senile or idiopathic xerosis, the favorable outcomes observed in this study suggest that the tested 10% urea lotion may also be beneficial in managing other dermatological conditions associated with skin dryness. These include diabetic dermopathy, atopic dermatitis (particularly in remission phases), and post‐menopausal skin, all of which are characterized by impaired barrier function and reduced hydration. Future studies could explore its application in these populations to further validate its therapeutic versatility. From a cosmetic dermatology perspective, these findings support the role of urea‐based dermocosmetic formulations as effective and well‐tolerated first‐line options for the management of xerosis in routine clinical and cosmetic practice.

### 4.1 Study Limitations

The limitations of this study include the absence of a control group (placebo or comparator), a relatively small sample size, and a short follow‐up period. Due to these factors, the results should be interpreted with caution; it is possible that a larger sample or longer follow‐up would provide additional insight into the long‐term effects and sustainability of improvements. Seasonal variations and environmental factors may have influenced skin hydration; however, standardized measurement conditions were applied to minimize their impact. Additionally, part of the evaluation relied on subjective patient reports (e.g., symptom ratings and satisfaction), which introduces the potential for reporting bias. Despite these limitations, the study offers valuable insight into the benefits of a 10% urea lotion for dry skin.

## 5 Conclusion

The results of this prospective clinical study clearly demonstrate that regular application of a 10% urea lotion enriched with Prolom thermal water significantly improves both objective and subjective parameters in individuals with xerosis. Over the 60‐day period, a statistically significant increase in skin hydration and elasticity was observed, along with reductions in symptoms such as dryness, itching, and scaling. In parallel, a substantial improvement in participants' quality of life, as measured by the Skindex‐16 questionnaire, was recorded, along with a high level of user satisfaction and excellent product tolerability. These findings are consistent with previous clinical trials of 10% urea formulations, confirming the efficacy and safety of this concentration in the treatment of dry skin.

## Author Contributions

Nemanja Maletin was responsible for the study conception and design, clinical implementation, data collection and analysis, interpretation of results, and drafting of the manuscript. Bojana Spasić, Zoran Golušin, and Ivana Binić provided supervision and coordination throughout all phases of the study. Miloš Nišavić handled study administration. Marija Ranđelović, Stefan Borocki, and Iva Binić contributed to study documentation and regulatory processes. Maša Golubović and Lazar Marin were responsible for patient management and follow‐up. Nikola Denda critically reviewed the manuscript and approved the final version. All authors read and approved the final manuscript.

## Funding

This research and the publication of this manuscript were funded by Planinka d.o.o., Prolom Banja, Serbia.

## Ethics Statement

The study was conducted in accordance with the ethical standards of the institutional research committees and with the 1964 Helsinki Declaration and its later amendments. Ethical approval was obtained from the Ethics Committee of the University Clinical Centre of Vojvodina, Clinic of Dermatovenereology (Approval No. 00‐156/2025; date: 21 March 2025) and the Ethics Committee of the Faculty of Medicine, University of Niš (Approval No. 12‐3102‐1/2‐6; date: 3 April 2025).

## Consent

All participants provided written informed consent prior to enrolment.

## Conflicts of Interest

This study was financially supported by Planinka d.o.o. *Prolom Banja*, which also covered the costs related to the publication of the scientific article. The sponsor had no role in the study design, data collection, data analysis, interpretation of results, or preparation of the manuscript.

## Data Availability

The data that support the findings of this study are available on request from the corresponding author. The data are not publicly available due to privacy or ethical restrictions.
